# Can CT Radiomics Predict the Ki-67 Index of Gastrointestinal Stromal Tumors (GISTs)? A Systematic Review and Meta-Analysis

**DOI:** 10.3390/cancers17172855

**Published:** 2025-08-30

**Authors:** Stavros P. Papadakos, Alexandra Argyrou, Ioannis Karniadakis, Charalampos Theocharopoulos, Ioannis Katsaros, Nikolaos Machairas, Jiannis Vlachogiannakos, Stamatios Theocharis

**Affiliations:** 1 First Department of Pathology, Medical School, National and Kapodistrian University of Athens, 11527 Athens, Greece; stavrospapadakos@gmail.com; 2 First Academic Department of Gastroenterology, Medical School of National and Kapodistrian University of Athens, General Hospital of Athens “Laiko”, 11527 Athens, Greece; argyalex89@gmail.com; 3 Upper Gastrointestinal Surgery, Department of General Surgery, St. George’s Hospital, St. George’s University Hospitals NHS Foundation Trust, London SW17 0QT, UK; ioanniskarniadakis@gmail.com; 4 Department of Surgery, University of Colorado Anschutz Medical Campus, Aurora, CO 80045, USA; hartheoch@gmail.com; 5 First Department of Surgery, National and Kapodistrian University of Athens, Laikon General Hospital, 11527 Athens, Greece; gikats.md@gmail.com; 6 Second Department of Propaedeutic Surgery, Laiko General Hospital, Schol of Medicine, National and Kapodistrian University of Athens, 11527 Athens, Greece; nmachair@med.uoa.gr

**Keywords:** gastrointestinal stromal tumors, CT radiomics, Ki-67 index, tumor proliferation, risk stratification

## Abstract

**Background/Objectives**: Computed tomography (CT)-based radiomic analysis is an emerging technique that enables non-invasive assessment of tumor characteristics. In gastrointestinal stromal tumors (GISTs), radiomics may reflect biological behavior such as proliferative activity, often indicated by Ki-67 expression. To our knowledge, this is the first systematic review and meta-analysis synthesizing evidence on the ability of CT radiomics to predict the Ki-67 index in GISTs, addressing an important gap in the literature. **Methods**: A systematic review and meta-analysis were conducted following PRISMA guidelines to evaluate the predictive performance of CT radiomics for Ki-67 expression in GISTs. A literature search of PubMed, Scopus, Science Direct, and the Cochrane Library was performed up to December 2024 using predefined terms. Extracted data included study design, patient demographics, imaging protocols, radiomic features, and diagnostic performance. Study quality was assessed using the QUADAS-2 tool. A random-effects meta-analysis summarized the pooled area under the ROC curve (AUC), sensitivity, and specificity. Subgroup and sensitivity analyses explored heterogeneity sources. Publication bias was assessed using Egger’s test and funnel plots. **Results**: Six studies involving 1632 patients were included. The pooled sensitivity and specificity for predicting Ki-67 expression were 0.71 and 0.76, respectively, with a summary AUC of 0.79. Subgroup analyses showed consistent results across different imaging protocols and radiomic feature sets, though the Ki-67 cutoff (8% vs. 10%) affected diagnostic performance. Moderate heterogeneity and potential publication bias in specificity were observed. **Conclusions**: CT-based radiomics demonstrates moderate accuracy for non-invasively predicting Ki-67 index in GISTs. While not a substitute for histology, it may support personalized preoperative planning and guide future immunotherapy strategies. In the future, radiomic signatures—particularly when integrated with molecular or immune-related biomarkers—could help refine patient selection and monitoring strategies for emerging therapies, including immunotherapy.

## Simple Summary

This systematic review and meta-analysis evaluated the potential of CT-based radiomics to non-invasively predict Ki-67 expression, a key marker of tumor proliferation, in gastrointestinal stromal tumors (GISTs). Six studies involving 1632 patients were analyzed. The pooled diagnostic performance of CT radiomics showed moderate accuracy, with a sensitivity of 0.71, specificity of 0.76, and an area under the ROC curve (AUC) of 0.79. Subgroup and sensitivity analyses confirmed consistent results across different imaging protocols and radiomic feature sets, although heterogeneity was noted, particularly in sensitivity. The choice of Ki-67 cutoff value (e.g., 8% vs. 10%) affected diagnostic performance. While radiomics is not a substitute for histopathology, it shows promise for preoperative risk assessment, offering a non-invasive insight into tumor heterogeneity and biology. The study recommends further standardization and suggests future research explore how radiomic features might help predict response to immunotherapy, supporting more personalized treatment strategies in GIST management.

## 1. Introduction

The mitotic count (MC), defined as the number of mitoses per 50 high-power fields, is a fundamental histopathological parameter in the evaluation and management of gastrointestinal stromal tumors (GISTs) [1]. As a marker of cellular proliferation, it plays a critical role in determining the malignant potential of GISTs and is incorporated into all major risk stratification models, including the National Institutes of Health (NIH) consensus and Armed Forces Institute of Pathology (AFIP) classifications [2,3]. Alongside tumor size, anatomical site, and rupture status, the mitotic index (MI) helps clinicians assess the likelihood of recurrence after surgical resection [4,5]. Beyond its current prognostic role, the MC may also prove valuable in shaping future therapeutic strategies.

Traditionally, MC has served as a prognostic marker. However, it may also help inform future therapies, including immunotherapy, by identifying biologically aggressive and immune-active tumors [6]. Additionally, emerging data indicate that certain preoperative CT imaging features, including size, necrosis, and tumor shape, show strong predictive value for the MI in GISTs [7], offering a non-invasive approach to guide risk stratification and individualized treatment planning.

The MC is a key parameter in evaluating the proliferative activity of GIST and plays a crucial role in risk stratification [4]; however, its accuracy can be limited by interobserver variability, as it relies heavily on the pathologist’s expertise and reflects only the M phase of the cell cycle [8]. To address these limitations, the Ki-67 labeling index has been increasingly utilized as a complementary marker, offering a broader assessment of tumor proliferation across all active phases except G0 and reducing variability through standardized immunohistochemical staining [9]. Recent evidence suggests that Ki-67 is a reliable surrogate marker for MC and may enhance preoperative risk stratification in GISTs [10,11,12,13]. Ki-67 expression—typically quantified as the percentage of positively stained tumor nuclei—has been validated as an independent prognostic marker in GISTs: values exceeding thresholds such as 5%, 6%, or 8% consistently associate with higher recurrence risk and shorter recurrence-free survival, even within traditional high-risk groups [14]. A recent large-sample meta-analysis confirmed that Ki-67 overexpression parallels increasing NIH risk categories and adds prognostic value beyond MC alone [15]. Additionally, Ki-67 scoring via digital or AI-assisted image analysis is emerging as a more reproducible and time-efficient alternative, reducing subjective bias and improving standardisation across laboratories [16].

Accurate preoperative assessment of tumor proliferative activity remains a critical unmet need in the management of GISTs, as it can help refine risk stratification and optimize treatment strategies. Radiomics is an advanced image analysis approach that leverages mathematical and computational algorithms to extract a large number of high-dimensional quantitative features from medical imaging modalities [17]. The typical workflow involves standardized image acquisition, tumor segmentation, feature extraction, and subsequent feature selection and modeling. Extracted features may include first-order statistics (intensity-based), shape descriptors, texture features that capture intratumoral heterogeneity, and higher-order features derived from filters or wavelet transformations. These quantitative descriptors can then be analyzed using machine learning or artificial intelligence techniques to identify robust imaging biomarkers. By converting sub-visual patterns into mineable data, radiomics provides a “virtual biopsy,” enabling reproducible characterization of tissue architecture that reflects underlying pathophysiological processes [18]. Beyond diagnosis, radiomics has been increasingly applied in oncology for prognosis, prediction of treatment response, and precision medicine. In the context of GISTs, CT-based radiomic analysis offers a non-invasive means to capture intratumoral heterogeneity, potentially serving as a surrogate for proliferative activity such as Ki-67 expression.

The objective of this study is to systematically review the current literature evaluating the utility of CT radiomics in predicting Ki-67 index in patients with GISTs, potentially enabling earlier identification of tumors with high proliferative potential.

## 2. Materials and Methods

This study was conducted in accordance with the Preferred Reporting Items for Systematic Reviews and Meta-Analyses (PRISMA) guidelines [19]. By following the PRISMA guidelines, we ensure transparency, reproducibility, and clarity in reporting, ultimately enhancing the quality and reliability of our findings.

A comprehensive search strategy was developed to identify relevant studies from four electronic databases, including PubMed, Scopus, ScienceDirect, and Cochrane Library. The search terms were carefully selected to ensure the inclusion of all relevant articles that met the predetermined eligibility criteria. Two independent reviewers conducted the initial screening of titles and abstracts, followed by a full-text assessment of potentially eligible articles. Any discrepancies were resolved through discussion.

The study protocol is registered with the PROSPERO international prospective register of systematic reviews (protocol number: CRD420251039199). This current meta-analysis utilized pre-existing published studies, obviating the need for ethical clearance or patient consent.

### 2.1. Search Strategy and Eligibility Criteria

A systematic online search of the PubMed, Scopus, Science Direct, and Cochrane Library databases was conducted until December 2024 to identify all articles regarding the prediction of Ki-67 expression in GISTs through CT analysis. The following search strategy was used for all databases: “gastrointestinal tumors” OR GISTs, AND “radiomics/machine learning/deep learning/artificial intelligence/texture” AND “computed tomography” OR CT, AND “Ki-67 antigen”. No date limit was used, and the search was performed up to December 2024 (see Supplementary Data S1) Additionally, the references for the identified articles were manually searched for additional studies.

### 2.2. Inclusion and Exclusion Criteria

The inclusion criteria for this systematic review and meta-analysis were as follows: (1) Study design: retrospective or prospective studies reporting original data and evaluating the diagnostic or predictive performance of CT radiomics for Ki-67 index prediction in GISTs, published in English. (2) Participants: patients of any age or sex with pathologically confirmed GISTs. (3) Index test: studies utilizing CT radiomics analysis, including any CT modality (non-contrast, contrast-enhanced, or multiphasic), and extracting radiomic features directly from GIST lesions. (4) Reference standard: pathological assessment of the Ki-67 index, either as a quantitative value or categorized level (e.g., high vs. low). (5) Outcomes: studies reporting diagnostic accuracy metrics such as sensitivity, specificity, AUC, or accuracy, as well as correlation or regression coefficients between radiomic features and Ki-67 index. Studies must provide sufficient data to calculate effect sizes or diagnostic accuracy parameters required for meta-analysis. Specifically, eligible studies were required to report at least one of the following: (a) sensitivity and specificity values (with or without confidence intervals); (b) area under the ROC curve (AUC); (c) accuracy; or (d) sufficient raw data (e.g., contingency tables of true positives, false positives, true negatives, and false negatives) from which these metrics could be derived. Studies not meeting these minimum data requirements were included only in the qualitative synthesis.

The exclusion criteria were (1) Study design: case reports, editorials, letters, reviews, and conference abstracts without full text, animal or in vitro studies, studies lacking original research, and those not published in English. (2) Participants: studies including patients with other gastrointestinal tumors or lacking pathological confirmation of GISTs. (3) Index test: studies that did not use CT radiomics or used other imaging modalities (e.g., MRI, PET, EUS) or failed to extract radiomic features from GISTs. (4) Reference standard: studies lacking pathological Ki-67 assessment or using surrogate markers. (5) Outcomes: studies not reporting relevant diagnostic or predictive outcomes, not providing sufficient data for meta-analysis, or showing significant unresolvable methodological flaws or bias. Such flaws were defined based on the Quality Assessment of Diagnostic Accuracy Studies tool (QUADAS-2 tool) as high risk of bias in multiple key domains (patient selection, index test, reference standard, or flow/timing), or methodological concerns that precluded reliable interpretation of results (e.g., unclear or inappropriate reference standard, inadequate reporting of index test methodology, or severe selection bias). (6) Duplicate publications: studies duplicating data already included elsewhere. (7) Overlapping populations: in cases of multiple studies with the same patient cohort, only the most complete and relevant study was retained. (8) Accessibility: studies without full-text availability. (9) Target outcome: studies using radiomics to predict endpoints unrelated to the Ki-67 index, such as mitotic index or overall malignancy risk.

### 2.3. Quality Assessment

Two researchers (AA and SP) independently evaluated the quality of case–control studies included in our meta-analysis using the QUADAS-2 tool [20]. The QUADAS-2 tool is used to assess the methodological quality of diagnostic accuracy studies, focusing on the risk of bias and applicability concerns across four key domains: patient selection, index test, reference standard, and flow and timing. Each domain is evaluated through specific signaling questions, helping to determine whether the study has a low, high, or unclear risk of bias. Additionally, concerns regarding the applicability of the study findings to the research question are assessed separately for the first three domains. A study is considered high-quality if it demonstrates a low risk of bias across all domains and minimal concerns regarding applicability. Discrepancies between the two investigators in the assessment were resolved through a reevaluation of the original article and discussion until consensus was reached.

### 2.4. Data Extraction and Synthesis

Data extraction was conducted using a standardized data collection form to ensure consistency across studies. Extracted data included study characteristics (author, year of publication, study design, sample size), participant demographics (age, gender, tumor characteristics), imaging protocols (scanner type, contrast phase), and radiomics methodology (feature extraction, machine learning models used). Specifically, for radiomics models, we recorded the feature extraction and selection methods, as well as the machine learning or statistical classifiers used. Across the included studies, models typically involved dimensionality reduction methods such as least absolute shrinkage and selection operator (LASSO) regression, followed by supervised learning approaches including logistic regression, random forest, or support vector machines. A subset of studies also explored deep learning–based models. This information was extracted to facilitate subgroup comparisons and to assess potential sources of heterogeneity in diagnostic performance. Outcome measures and effect sizes were also systematically recorded.

To minimize potential double-counting of patients, we carefully examined the study settings, timeframes, and authorship of included articles to identify possible overlapping cohorts. In cases where overlap was suspected, the study with the larger sample size or more complete dataset was prioritized for inclusion. No confirmed overlapping populations were identified.

For the meta-analysis, the area under the receiver operating characteristic curve (AUC) was used as the primary effect size to evaluate the predictive performance of CT radiomics in distinguishing high from low Ki-67 expression in gastrointestinal stromal tumors (GISTs) [21]. Additional diagnostic performance metrics, including sensitivity, specificity, and accuracy, were extracted where available. In cases where studies did not report these values directly, they were derived from contingency tables provided in the original data.

A random-effects meta-analysis was performed using the generic inverse variance method to synthesize AUC values across studies. Heterogeneity among included studies was assessed using Cochran’s Q test and the I^2^ statistic, with substantial heterogeneity defined as I^2^ > 50% or *p* < 0.10 [22]. Sensitivity and subgroup analyses were conducted to explore potential sources of heterogeneity, including differences in the quality of studies, CT imaging protocols, radiomics feature selection, and Ki-67 index cutoff.

A bivariate random-effects meta-analysis model was initially considered for pooling sensitivity and specificity. However, due to the limited number of included studies (n = 6) and inconsistent reporting of contingency data across articles, model convergence could not be reliably achieved. Therefore, we used separate random-effects models for sensitivity and specificity, which is an accepted approach in small-sample diagnostic accuracy meta-analyses.

To evaluate study quality and risk of bias, we applied the QUADAS-2 tool for diagnostic accuracy studies. Publication bias was assessed through Egger’s test and funnel plot asymmetry analysis. All statistical analyses were conducted using IBM SPSS Statistics 29.0.1.0 and R package 4.4.3 for Windows. SPSS was primarily used for descriptive statistics and initial data management, while R was used for the meta-analysis. In R, pooled sensitivity, specificity, and AUC were calculated using random- or fixed-effects models through the “meta” and “mada” packages, and forest plots, summary receiver operating characteristic (SROC) curves, and funnel plots were generated with the “metafor” package. Meta-analysis results were presented as pooled estimates with 95% confidence intervals (CIs), with statistical significance set at *p* < 0.05 for all analyses except heterogeneity assessments, where a threshold of *p* < 0.10 was used.

### 2.5. Sensitivity Analysis

A sensitivity analysis was conducted to assess the influence of excluding specific studies from the meta-analysis, considering the weight assigned to each study, excluding studies with a large weight, and considering the outcomes of the critical evaluation of individual studies.

The sensitivity analysis also incorporated the findings of the QUADAS-2 assessment, which evaluates four key domains: patient selection, index test, reference standard, and flow and timing, each assessed for risk of bias and applicability concerns.

### 2.6. Subgroup Analysis

Considering the significant heterogeneity observed among the studies, it was deemed necessary to perform subgroup analyses. These analyses were conducted between studies rather than within individual datasets. Studies were stratified according to reported cohort type (training vs. validation cohorts when separately published), CT imaging protocol (non-contrast, arterial, venous), number of radiomics features examined (<800 vs. ≥800), and Ki-67 index cutoff (8% vs. 10%). By performing these between-study subgroup analyses, we aimed to identify potential methodological and clinical factors that may have influenced diagnostic performance, while avoiding misinterpretation as intra-study pooling.

### 2.7. Publication Bias Risk Assessment

In the absence of publication bias, the funnel plot resembled an inverted funnel, with smaller studies scattered widely at the bottom and larger studies clustering near the top [23,24].

## 3. Results

### 3.1. Literature Search

**Figure 1:**
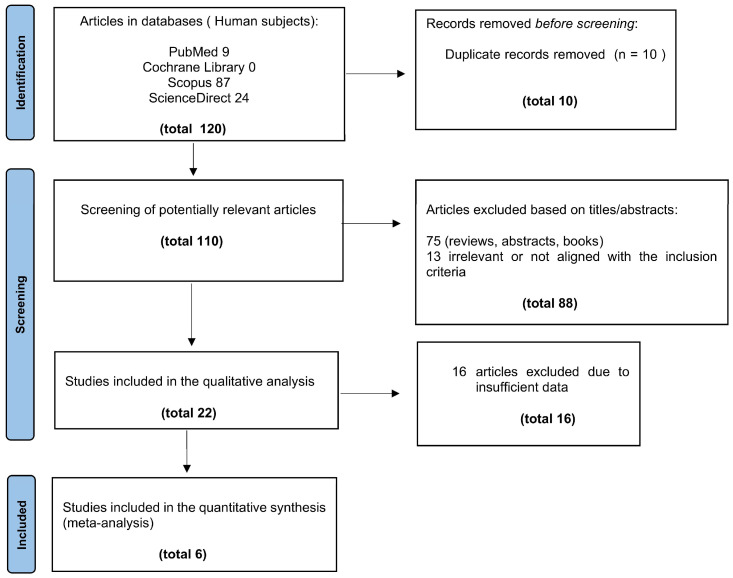
Flowchart of the study selection procedure.

The search strategy yielded a total of 120 articles. After removing duplicates, 110 unique studies remained for title and abstract screening. Of these, 83 articles were excluded as not congruent with the eligibility criteria. Six eligible studies were identified [25,26,27,28,29,30], after full text screening and application of the inclusion/exclusion criteria, and the individual references listed in each publication were further investigated for ascertainment of additional cases. The search strategy is presented in Figure 1.

### 3.2. Quality Assessment

**Figure 2:**
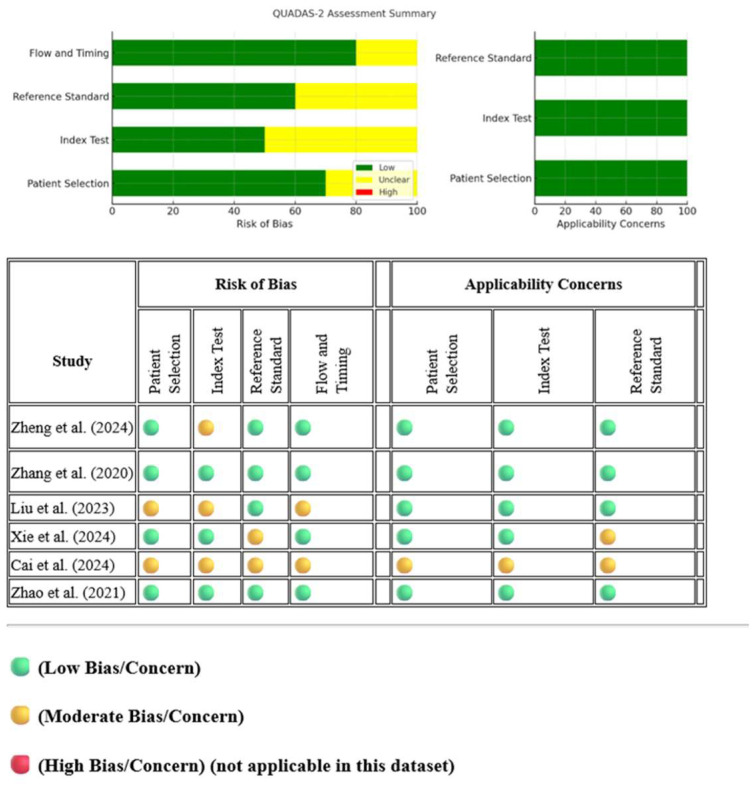
Methodologic Quality Assessment of the Studies Based on the QUADAS-2 Scale. The references were cited in the figure.

QUADAS-2 evaluation identified a moderate risk of bias in certain studies [25,26,27,28], particularly in the domains of patient selection, index test, and reference standard. These findings suggest potential sources of heterogeneity within the meta-analysis, emphasizing the need for careful consideration of study quality in the interpretation of pooled results, as illustrated in Figure 2.

### 3.3. Main Findings

All included studies reported data on the precise number, gender, and mean age of patients, with the exception of two studies that did not provide information on patient age and gender [25,26,27,28]. In total, 1632 patients were included in the present study, while 774 (47.4%) were females, and the mean age ranged from 58, 96 to 61, 96 years [25,26,27,28,29,30].

Furthermore, all studies documented the tumor type (GISTs), details regarding the CT scanner used, the number of radiomics features analyzed, and the machine learning models employed. Additionally, five studies reported the CT contrast phase [25,26,28,29,30], and the cutoff value for Ki-67 antigen [25,26,27,29,30].

### 3.4. Pooled Analysis of Diagnostic Performance

To ensure that each study is represented only once in the meta-analysis and to appropriately group the results, it is crucial to aggregate effect sizes at the study level rather than treating different cohorts (training cohort, internal validation cohort, external validation cohort), machine learning models, or imaging phases as separate entries. This prevents redundancy and ensures a more accurate estimation of study-level effect sizes. When multiple effect sizes (e.g., AUC, sensitivity, specificity) are reported within the same study due to the above variations, a weighted average must be computed before inclusion in the meta-analysis. By aggregating effect sizes at the study level, the analysis more accurately reflects the overall impact of each study while accounting for within-study variability. This approach mitigates potential biases arising from the overrepresentation of studies reporting multiple results under different conditions, ensuring methodological consistency and improving the interpretability and reliability of the findings.

**Figure 3:**
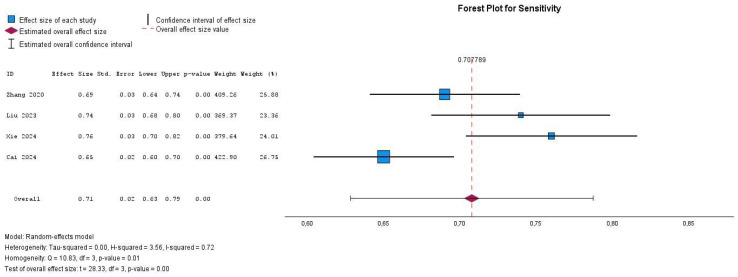

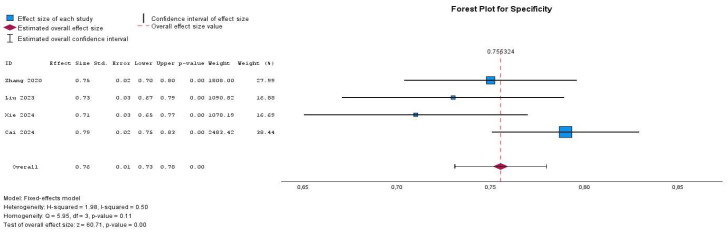
Forest plot illustrating the diagnostic performance of CT radiomics in predicting the Ki-67 index in GISTs, based on sensitivity and specificity from individual studies. The studies are presented in chronological order. Studies are listed in chronological order.

The sensitivity and specificity of the included studies in the meta-analysis varied across different cohorts. Out of the six eligible studies, four were incorporated into the analysis due to available data on diagnostic performance (sensitivity, specificity). The sensitivity estimates ranged from 0.65 to 0.76, while specificity estimates ranged from 0.71 to 0.79. The pooled sensitivity was 0.71 (95% CI, 0.63–0.79), and the pooled specificity was 0.76 (95% CI, 0.73–0.78), as illustrated in Figure 3.

The forest plots for sensitivity and specificity indicate the degree of heterogeneity across the included studies. For sensitivity, the I^2^ statistic is 72%, with a significant Q-test (Q = 10.83, *p* = 0.01), suggesting substantial heterogeneity among the studies. In contrast, the specificity analysis shows an I^2^ value of 50%, with a Q-test of 5.95 (*p* = 0.11), indicating moderate heterogeneity. These findings suggest that variability among studies may be influenced by differences in study design, patient populations, or diagnostic thresholds. A random-effects model was appropriately applied to account for this heterogeneity.

**Figure 4:**
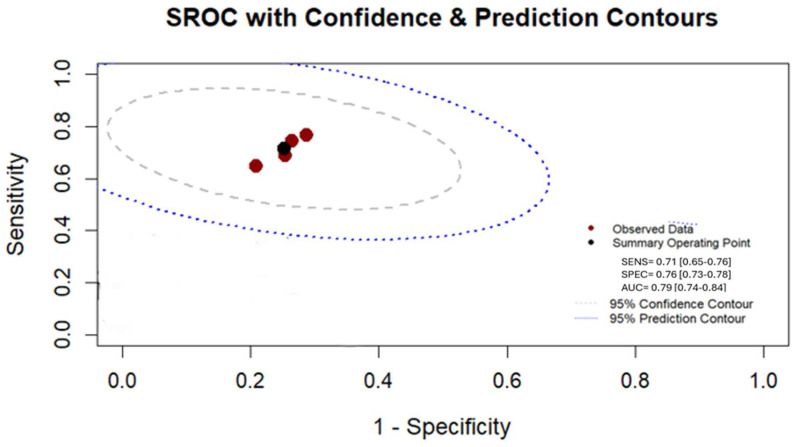
Summary receiver operating characteristic (SROC) plot illustrating the performance of CT radiomics in predicting the Ki-67 index in GISTs.

The calculated positive likelihood ratio (PLR), negative likelihood ratio (NLR), and diagnostic odds ratio (DOR) were 2.83 (95% CI, 2.64–3.35), 0.39 (95% CI, 0.33–0.46), and 7.32 (95% CI, 6.17–8.67), respectively. Furthermore, the summary receiver operating characteristic (SROC) curve is depicted in Figure 4, with the pooled area under the curve (AUC) of the included studies estimated at 0.79 (95% CI, 0.74–0.84). (Table 1).

### 3.5. Sensitivity Analysis

**Figure 5:**
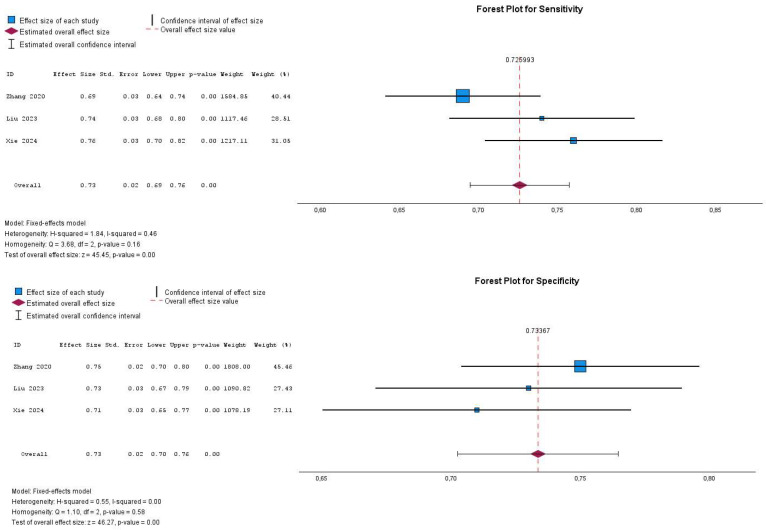
Forest plot demonstrating the pooled diagnostic performance (sensitivity and specificity) of CT radiomics in predicting the Ki-67 index of GISTs after excluding the study with the greatest weight. Studies are listed in chronological order.

Through a sensitivity analysis that considered the weight of each study, one study conducted by Cai et al. was removed as it carried the most significant weight in the meta-analysis results [27]. Following the exclusion of a highly weighted study, the meta-analysis resulted in a pooled sensitivity of 0.73 (95% CI: 0.69–0.76, *p*-value < 0.001) and a pooled specificity of 0.73 (95% CI: 0.70–0.76, *p*-value < 0.001), indicating that CT radiomics has a moderate ability to predict the Ki-67 index in GISTs. The heterogeneity in the sensitivity analysis was moderate (I^2^ = 46%, Cochrane Q test = 3.68, *p*-value = 0.16), indicating some variability among studies, although without statistically significant results, whereas the specificity analysis exhibited no heterogeneity (I^2^ = 0%, Cochrane Q test = 1.10, *p*-value = 0.58), suggesting a high level of consistency across studies. These findings highlight the robustness of the specificity estimates while indicating that the removed study had a notable impact on the sensitivity analysis, contributing to the observed heterogeneity, as illustrated in Figure 5 (Table 1).

**Figure 6:**
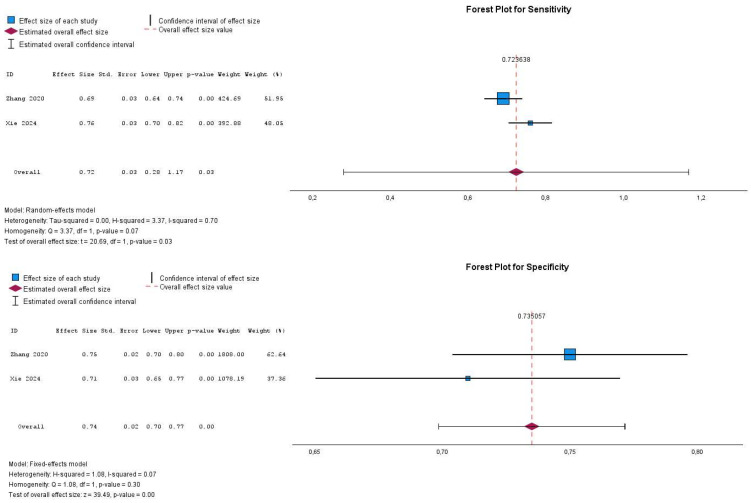
Forest plot demonstrating the pooled diagnostic performance (sensitivity and specificity) of CT radiomics in predicting the Ki-67 index of GISTs after excluding the studies with poor/fair quality according to the QUADAS-2 tool. Studies are listed in chronological order.

The QUADAS-2 assessment revealed variations in the methodological quality of the six included studies. While most studies exhibited a low risk of bias in patient selection, the studies of Liu et al. [25] and Cai et al. [27] demonstrated a moderate risk, likely due to potential selection biases within their cohorts. In the index test domain, Zheng et al. [28], Liu et al. [25], and Cai et al. [27] showed a moderate risk of bias, possibly resulting from differences in feature extraction methods or the machine learning models employed. Regarding the reference standard, the majority of studies maintained a low risk of bias; however, Xie et al. [26] and Cai et al. [27] exhibited a moderate risk, which may be attributed to inconsistencies in Ki-67 assessment methodologies. Lastly, the flow and timing domain was generally associated with a low risk of bias, although Liu et al. [25] and Cai et al. [27] demonstrated a moderate risk, potentially due to variations in imaging protocols or delays in histopathologic validation. After performing a sensitivity analysis by excluding the two lower-quality studies (Liu et al. and Cai et al.) (the studies of Zheng et al. [28] and Zhao et al. [30] were not included since the beginning of the meta-analysis due to lack of data), the pooled specificity and sensitivity remained relatively stable, as reflected in the forest plots. The specificity analysis demonstrated low heterogeneity (I^2^ = 7%, Cochrane Q test = 1.08, *p*-value = 0.30), suggesting consistency among the remaining studies in assessing CT radiomics’ ability to predict the Ki-67 index in GISTs. However, the sensitivity analysis exhibited a higher degree of heterogeneity (I^2^ = 70%, Cochrane Q test = 3.37, *p*-value = 0.07), indicating greater variability among the included studies in terms of sensitivity estimation. This increased heterogeneity may be attributed to variations in imaging protocols, feature extraction methods, or differences in histopathologic validation across studies. Additionally, the exclusion of the two lower-quality studies may have influenced the balance of methodological approaches, leading to greater disparities in sensitivity outcomes. Despite these differences, the results reinforce the diagnostic potential of CT radiomics while highlighting the need for standardization in radiomic feature extraction and validation methodologies, as illustrated in Figure 6. (Table 1).

### 3.6. Subgroup Analysis

#### 3.6.1. By Type of Cohort

A subgroup analysis by cohort was conducted to evaluate the predictive performance of CT radiomics for the Ki-67 index in GISTs, considering both sensitivity and specificity. The sensitivity analysis demonstrated an overall pooled sensitivity of 0.74 (95% CI: 0.73–0.75, *p*-value < 0.01). Across the training and validation cohorts, the sensitivity values remained consistent, with minimal variability observed between subgroups (test of between-subgroup homogeneity: *p* = 0.95). Similarly, the specificity analysis indicated an overall pooled specificity of 0.73 (95% CI: 0.72–0.74, *p*-value < 0.01), showing comparable performance across different cohorts (test of between-subgroup homogeneity: *p* = 1.00). Notably, heterogeneity was observed in sensitivity (I^2^ = 46%) but was lower for specificity (I^2^ = 3%), suggesting a more stable specificity estimate across studies. The consistency of CT radiomics in predicting the Ki-67 index across diverse study cohorts is strongly supported by these results (see Supplementary Data S2) (Table 1).

#### 3.6.2. By CT Imaging Protocols

By CT imaging protocols: A subgroup analysis was conducted based on different CT imaging phases used in each study. The studies were stratified into non-contrast, arterial, venous, and delayed phases. The pooled sensitivity for predicting the Ki-67 index across all CT phases was 0.75 (95% CI: 0.74–0.77), with no significant between-subgroup heterogeneity (I^2^ = 0%, Cochrane Q test = 24.42, *p*-value = 0.96). Similarly, the pooled specificity across CT imaging protocols was 0.73 (95% CI: 0.72–0.74), with no significant subgroup effect (I^2^ = 0%, Cochrane Q test = 7.54, *p*-value = 1.00). The findings indicate that the predictive performance of CT radiomics for the Ki-67 index in GISTs remains consistent across different imaging protocols, supporting its robustness regardless of the phase of image acquisition (see Supplementary Data S2) (Table 1).

#### 3.6.3. By the Number of Radiomics Features

In a subgroup analysis stratified by the number of radiomic features employed in each study, utilizing a cutoff of 800 features, the diagnostic performance of CT radiomics in predicting the Ki-67 index for GISTs was assessed. Studies employing fewer than 800 features demonstrated a pooled sensitivity of 0.76 (95% CI: 0.75–0.77) and a pooled specificity of 0.71 (95% CI: 0.70–0.72), whereas studies utilizing 800 features or more exhibited a pooled sensitivity of 0.72 (95% CI: 0.70–0.74) and a pooled specificity of 0.76 (95% CI: 0.74–0.76). While there was a numerical difference observed in pooled sensitivity and specificity, the subgroup effect was statistically significant for specificity (Q = 15.81, df = 1, *p* < 0.001), indicating a potential influence of feature count on diagnostic performance. Within-subgroup heterogeneity was negligible for specificity (I^2^ = 0%, Cochrane Q test =3 4.28, *p*-value = 0.76) across both subgroups, suggesting consistency in specificity within each feature count category. However, for sensitivity, within-subgroup heterogeneity was moderate in both subgroups (I^2^ = 46%, Cochrane Q test = 74.10, *p*-value < 0.01), indicating variability in sensitivity within each feature count category (see Supplementary Data S2) (Table 1).

#### 3.6.4. By the Ki-67 Index Cutoff

To examine the influence of Ki-67 index cutoff values on the diagnostic efficacy of CT radiomics in predicting Ki-67 expression in GISTs, a subgroup analysis was conducted, stratifying studies based on the applied cutoff value (8% or 10%). Studies utilizing a Ki-67 index cutoff of 8% reported a pooled sensitivity of 0.74 (95% CI: 0.73–0.75) and a pooled specificity of 0.77 (95% CI: 0.76–0.79). Conversely, studies employing a Ki-67 index cutoff of 10% demonstrated a pooled sensitivity of 0.76 (95% CI: 0.75–0.78) and a pooled specificity of 0.71 (95% CI: 0.67–0.76). Despite observed numerical variations in pooled sensitivity and specificity across subgroups, the subgroup effect was statistically significant for both sensitivity (Q = 34.72, df = 1, *p*-value < 0.001) and specificity (Q = 17.57, df = 1, *p*-value < 0.001), indicating that the Ki-67 index cutoff utilized in individual studies significantly impacts the diagnostic performance of CT radiomics. Within-subgroup heterogeneity for specificity was negligible across both cutoff categories (I^2^ = 0.00%, Cochrane Q test = 34.28, *p*-value = 0.76), signifying consistency in specificity within each subgroup. However, for sensitivity, substantial within-subgroup heterogeneity was observed in studies using a Ki-67 cutoff of 10 (I^2^ = 74.10%, Cochrane Q test = 74.10, *p*-value < 0.001), while heterogeneity was negligible in studies employing a Ki-67 cutoff of 8% (I^2^ = 0%, Cochrane Q test *p*-value = 0.73) (see Supplementary Data S2) (Table 1).

### 3.7. Publication Bias

**Figure 7:**
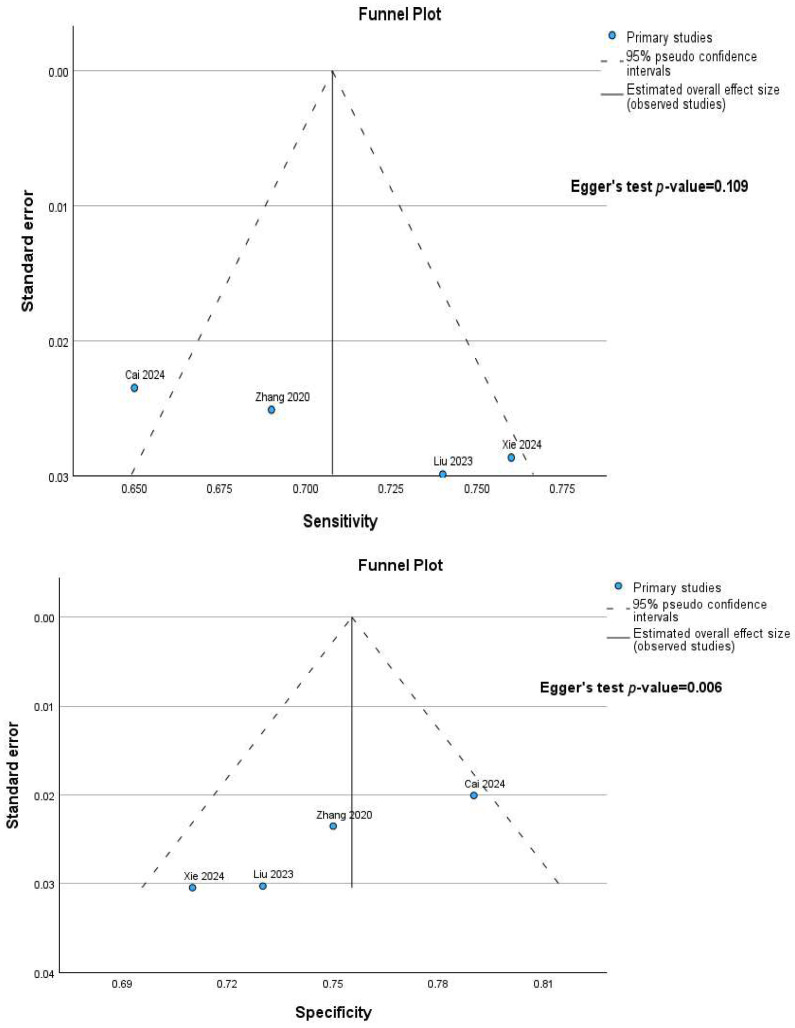
Funnel Plot and Egger’s Regression Test of the pooled diagnostic performance (sensitivity, specificity) of the studies included in the meta-analysis.

To assess potential publication bias within this meta-analysis, funnel plots and Egger’s regression tests were conducted for both sensitivity and specificity. Visual inspection of the sensitivity funnel plot indicated a relatively symmetrical distribution of studies, and Egger’s regression test yielded a non-significant intercept (*p* = 0.109), suggesting a low likelihood of publication bias affecting sensitivity results. Conversely, for specificity, while the funnel plot also presented a relatively symmetrical distribution, Egger’s regression test demonstrated a statistically significant intercept (*p* = 0.006), indicating potential asymmetry and raising concerns for publication bias. Specifically, this suggests a possibility of unpublished studies with lower specificity values. It is important to acknowledge that the limited number of studies in this meta-analysis restricts the power of these tests and the reliability of visual funnel plot interpretation. Therefore, while Egger’s test suggests potential bias in specificity, these findings should be interpreted cautiously. Nonetheless, the results indicate a need for careful consideration of publication bias, particularly when interpreting the specificity findings of this meta-analysis. Figure 7 depicts a consistent pattern of results that was observed in the funnel plots generated for the majority of subgroup analyses conducted.

## 4. Discussion

Our systematic review and meta-analysis investigated the diagnostic accuracy of CT-based radiomic models in predicting the Ki-67 proliferation index in patients with GISTs. A total of 6 studies, encompassing 1632 patients, were included and analyzed. The pooled sensitivity and specificity of CT radiomics for identifying high Ki-67 expression were 0.71 (95% CI: 0.63–0.79) and 0.76 (95% CI: 0.73–0.78), respectively, with a summary AUC of 0.79 (95% CI: 0.74–0.84), indicating moderate diagnostic performance. These findings are in line with prior research suggesting a correlation between radiomic features and markers of tumor aggressiveness, including Ki-67 expression [10,29].

Subgroup analyses demonstrated consistent radiomic performance across various CT imaging phases (non-contrast, arterial, venous), feature extraction thresholds (<800 vs. ≥800 features), and study cohorts (training vs. validation sets), suggesting robustness of the models across technical variations. However, diagnostic sensitivity and specificity varied significantly with different Ki-67 index cutoffs, as studies using a 10% threshold showed higher sensitivity but lower specificity than those using an 8% threshold [25,27]. This finding underscores the urgent need for consensus on standardized cutoff values to improve comparability across studies and facilitate clinical translation.

Sensitivity analyses confirmed that exclusion of high-weight or lower-quality studies did not significantly alter pooled diagnostic metrics, although heterogeneity remained notable, particularly in sensitivity (I^2^ = 72%). This degree of unexplained heterogeneity indicates that differences in CT acquisition protocols, segmentation approaches, feature extraction pipelines, machine learning algorithms, and Ki-67 thresholds substantially influenced model performance across studies. Consequently, the generalizability of our pooled estimates is limited, and the findings should be interpreted with caution until validated in more standardized, prospective multicenter settings.

Radiomics methodology itself lacks standardization, with wide variation in segmentation techniques, software platforms, and feature definitions. Moreover, harmonization strategies such as ComBat, which reduce scanner-related variability, were rarely reported, further compromising reproducibility. Establishing standardized radiomics pipelines with harmonized acquisition, segmentation, and feature extraction methods will be crucial for progress in this field.

The pooled diagnostic accuracy observed in our meta-analysis (AUC 0.79) suggests that CT-based radiomics holds promise as a non-invasive adjunct in the preoperative assessment of GISTs. However, the performance remains moderate compared with histopathology, reinforcing that radiomics should currently be considered complementary rather than substitutive. Notably, the consistency of results across imaging phases and feature extraction thresholds supports the technical robustness of radiomics, although heterogeneity in Ki-67 cutoff definitions (8% vs. 10%) significantly affected diagnostic outcomes. This finding highlights the urgent need for consensus regarding threshold selection in order to improve comparability across studies. When contrasted with radiomics applications in other solid tumors, such as lung or liver cancers, where pooled AUCs frequently exceed 0.85, the performance in GISTs appears slightly lower, likely reflecting both biological complexity and the relative scarcity of available datasets. Nevertheless, our results establish a foundation for integrating radiomics into existing prognostic models, potentially enhancing the precision of preoperative risk stratification.

It is important to note, however, that while Ki-67 has been validated as a prognostic biomarker in GIST and correlates with overall and disease-free survival, it is not currently incorporated into standard risk stratification systems, which continue to rely primarily on mitotic count. As such, radiomics models developed to predict Ki-67 should be interpreted with caution. Without strong prospective evidence directly linking radiomics-based Ki-67 prediction to patient outcomes, these models cannot yet be considered ready for routine clinical adoption. Instead, they may serve as exploratory tools that complement—but not replace—established risk factors, generating hypotheses for future validation in large, outcome-driven cohorts. This is particularly important given that most included studies were retrospective, often single-center, and lacked external validation, which further limits the generalizability of current findings. Ultimately, the clinical utility of such radiomics models will depend on whether they demonstrate added prognostic or predictive value beyond mitotic count and other established variables in prospective, multicenter studies.

While Ki-67 is increasingly recognized as a complementary marker reflecting proliferation across all active phases of the cell cycle except G0, and has been shown to correlate with mitotic rate, which is considered the gold standard for prognostication in GISTs [2,3,10], its standalone prognostic value necessitates further investigation. In a large meta-analysis of 1967 patients with GISTs from 24 studies, high Ki-67 expression was significantly correlated with worse overall survival (HR: 3.73; 95%CI: 2.81–4.93, *p* < 0.001) and worse disease-free survival (HR: 3.65; 95%CI: 2.68–4.97, *p* < 0.001) [31]. Nevertheless, our findings support the role of CT radiomics as a non-invasive tool for preoperative risk stratification, especially in cases where biopsy or full histologic evaluation is not feasible due to tumor location or surgical unresectability. A key advantage of radiomics over biopsy lies in its ability to capture the entire tumor landscape, rather than a limited tissue sample of a few millimeters, thereby offering a more comprehensive assessment of spatial heterogeneity. As the field evolves, a promising future perspective includes efforts to identify radiomic signatures predictive of response to immune checkpoint inhibitors, potentially guiding neoadjuvant immunotherapy in selected GIST patients and further advancing precision treatment strategies.

Emerging evidence suggests that radiomics holds substantial promise in transforming the diagnostic and therapeutic landscape of GISTs. Zhang et al. demonstrated that CT-based radiomic features can be used to infer KIT exon 11 mutation status, offering a non-invasive method to support genotype-driven therapy selection [32]. Similarly, Xie et al. highlighted the potential of radiomics in identifying PDGFRA D842V mutations, which are resistant to standard TKIs, enabling earlier therapeutic redirection [33]. Wang et al. emphasized the prognostic relevance of radiomics by linking imaging-derived features to recurrence-free survival, suggesting their utility in postoperative surveillance planning [34]. Ji et al. introduced delta-radiomics for the prediction of tumor recurrence within three years after surgery, providing a dynamic framework for longitudinal monitoring [35]. Additionally, Yang et al. demonstrated the integration of radiomics with deep learning to estimate the mitotic index, moving toward automated and histopathology-independent risk assessment [36]. Collectively, these findings underscore the expanding role of radiomics in not only diagnostic and prognostic modeling but also in capturing the molecular and proliferative landscape of GISTs. Integration of radiomic features with clinical and genomic data is likely to yield more powerful and clinically relevant models, supporting the development of personalized risk stratification and treatment strategies.

This study has several limitations that should be acknowledged. Firstly, although the Ki-67 index is a widely accepted marker of tumor proliferation, it is the mitotic count—not Ki-67—that is directly incorporated into established GIST prognostic models and risk stratification systems. Therefore, the use of Ki-67 as a surrogate may not fully capture the prognostic implications of proliferative activity. Secondly, the limited number of eligible studies (n = 6) and patients (1632 in total) reduces the statistical power and may limit the generalizability of the findings. Moreover, most included studies originated from single institutions or specific geographic regions, which further constrains external validity. Thirdly, substantial heterogeneity was observed in imaging protocols, feature extraction pipelines, Ki-67 cutoff thresholds, and machine learning approaches, which may have influenced pooled estimates. In addition, radiomics methodology itself remains heterogeneous, with differences in segmentation techniques, software platforms, and feature definitions not always reported in sufficient detail, limiting reproducibility. Importantly, radiomic features are known to be sensitive to variations in image acquisition and reconstruction; however, reporting on the use of harmonization methods (e.g., ComBat) was limited or absent across the included studies. This lack of standardization and reporting may have contributed to the observed heterogeneity and highlights the need for future research to explicitly apply and document harmonization strategies to improve reproducibility. Fourthly, variability in methodological quality was noted, with moderate risk of bias in domains such as patient selection, index test, and reference standard in several studies, as assessed by the QUADAS-2 tool. Although subgroup and sensitivity analyses helped explore sources of heterogeneity, potential publication bias—particularly for specificity—was indicated by Egger’s test, raising the possibility that lower-performing studies may be underrepresented in the literature. Furthermore, the retrospective design of most studies and the absence of external validation restrict the robustness of the results. There is also a risk of model overfitting in radiomics analyses, especially in studies with relatively small cohorts and high-dimensional feature sets, which may compromise generalizability to independent populations. Although internal validation strategies were often used, external validation was largely absent, limiting confidence in generalizability. Future studies should adopt rigorous approaches such as nested cross-validation, penalization methods, and independent external cohorts to ensure model robustness. Lastly, while radiomics shows promise, its clinical utility in routine GIST management remains exploratory at present. These limitations highlight the need for standardized, prospective multicenter studies, harmonized radiomics pipelines, transparent reporting of software and feature extraction methods, and integration with clinical and genomic data to validate and establish CT-based Ki-67 prediction in clinical practice. Standardized, prospective multicenter studies, coupled with publicly accessible imaging repositories for reproducibility and external validation, will be essential to move the field forward.

## 5. Conclusions—Future Perspectives

This systematic review and meta-analysis demonstrate that CT-based radiomic analysis shows moderate accuracy in predicting the Ki-67 proliferation index in patients with GISTs, with consistent diagnostic performance across diverse imaging protocols and study settings. While Ki-67 is not yet a standalone prognostic biomarker, its radiomic prediction offers a non-invasive alternative to histopathology, especially when biopsy is limited or unfeasible. Radiomics may capture the full spatial heterogeneity of the tumor, offering advantages over conventional standard biopsy sampling. However, its clinical utility will ultimately depend on demonstrating added value beyond mitotic count, and on prospective evidence linking radiomics-based Ki-67 prediction to patient outcomes. Future research should prioritize multicenter validation, harmonization of imaging protocols and feature extraction pipelines, and integration with clinical and genomic data. Equally important will be the development of publicly accessible multicenter imaging repositories, which can facilitate data sharing, reproducibility, and external validation across diverse patient populations and imaging platforms. These advancements may ultimately establish radiomics as a central tool in the personalization of GIST management, particularly in guiding emerging therapeutic strategies such as administration of neoadjuvant immunotherapy. Only through prospective, multicenter, outcome-driven studies, supported by standardized and reproducible radiomics pipelines, can CT-based radiomics be advanced from an exploratory research tool to a clinically applicable adjunct in GIST management.

## Figures and Tables

**Table 1:** Results of meta-analyses by type of outcome.

Outcome of Interest	Number of Studies	Effect Size (95% CI)	Effect Model		Heterogeneity	
				I^2^	*p*-value	Q-statistic
*Prediction of Ki-67 index of GISTs through CT radiomics*						
Pooled AUC	6	0.79 (0.74–0.84)	Fixed	0%	0.99	0.15
Pooled Sensitivity	4	0.71 (0.63–0.79)	Random	72%	0.01	10.83
Pooled Specificity	4	0.76 (0.73–0.78)	Fixed	50%	0.11	5.95
(sensitivity analysis after excluding large studies)						
Pooled Sensitivity	2	0.73 (0.69–0.76)	Fixed	46%	0.16	3.68
Pooled Specificity	2	0.73 (0.70–0.76)	Fixed	0%	0.58	1.10
(sensitivity analysis after excluding low-quality studies)						
Pooled Sensitivity	2	0.72 (0.28–1.17)	Random	70%	0.07	3.37
Pooled Specificity	2	0.74 (0.70–0.77)	Fixed	7%	0.30	1.08
(subgroup analysis by type of cohort)						
Pooled Sensitivity	4	0.74 (0.73–0.77)	Random	46%	<0.01	74.10
Pooled Specificity	4	0.73 (0.73–0.74)	Fixed	0%	0.76	34.28
(subgroup analysis by CT imaging protocols)						
Pooled Sensitivity	3	0.75 (0.74–0.76)	Fixed	0%	0.96	24.41
Pooled Specificity	3	0.73 (0.72–0.74)	Fixed	0%	1.00	7.54
(subgroup analysis by number of radiomics features)						
Pooled Sensitivity	4	0.74 (0.73–0.75)	Random	46%	<0.01	74.10
Pooled Specificity	4	0.73 (0.73–0.74)	Fixed	0%	0.76	34.28
(subgroup analysis by Ki-67 index cutoff)						
Pooled Sensitivity	4	0.74 (0.73–0.74)	Random	46%	<0.01	74.10
Pooled Specificity	4	0.73 (0.73–0.74)	Fixed	0%	0.76	34.28
CI, confidence interval;						

## Supplementary Materials

The following supporting information can be downloaded from https://www.mdpi.com/article/10.3390/cancers17172855/s1.
