# Supplementary material for: Can CT Radiomics Predict the Ki-67 Index of Gastrointestinal Stromal Tumors (GISTs)? A Systematic Review and Meta-Analysis
Source: Cancers (Basel). 2025 Aug 30;17(17):2855. doi: 10.3390/cancers17172855 (PMC12427292; doi:10.3390/cancers17172855)
Supplement: Supplementary file 1 [file cancers-17-02855-s001.zip › cancers-3788164_SUPPLEMENTARY DATA.pdf]

SUPPLEMENTARY DATA

S1: SEARCH STRING IN PUBMED

(("radiomics"[MeSH Terms] OR "radiomics"[All Fields] OR "radiomic"[All Fields] OR (("textural"[All Fields] OR "texturally"[All Fields] OR "texture"[All Fields] OR "texture s"[All Fields] OR "textured"[All Fields] OR "textures"[All Fields] OR "texturing"[All Fields] OR "texturization"[All Fields] OR "texturize"[All Fields] OR "texturized"[All Fields] OR "texturizing"[All Fields]) AND ("analysis"[MeSH Subheading] OR "analysis"[All Fields])) OR (("quantitate"[All Fields] OR "quantitated"[All Fields] OR "quantitates"[All Fields] OR "quantitating"[All Fields] OR "quantitation"[All Fields] OR "quantitations"[All Fields] OR "quantitative"[All Fields] OR "quantitatively"[All Fields] OR "quantitativeness"[All Fields] OR "quantitatives"[All Fields] OR "quantitive"[All Fields] OR "quantitively"[All Fields]) AND ("image"[All Fields] OR "image s"[All Fields] OR "imaged"[All Fields] OR "imager"[All Fields] OR "imager s"[All Fields] OR "imagers"[All Fields] OR "images"[All Fields] OR "imaging"[All Fields] OR "imaging s"[All Fields] OR "imagings"[All Fields]))) AND ("gastrointestinal stromal tumors"[MeSH Terms] OR ("gastrointestinal"[All Fields] AND "stromal"[All Fields] AND "tumors"[All Fields]) OR "gastrointestinal stromal tumors"[All Fields] OR ("gastrointestinal"[All Fields] AND "stromal"[All Fields] AND "tumor"[All Fields]) OR "gastrointestinal stromal tumor"[All Fields] OR ("gastrointest stromal tumor"[Journal] OR "gist"[All Fields])) AND ("ki 67"[All Fields] OR ("mitotic index"[MeSH Terms] OR ("mitotic"[All Fields] AND "index"[All Fields]) OR "mitotic index"[All Fields] OR ("proliferation"[All Fields] AND "index"[All Fields]) OR "proliferation index"[All Fields]))) AND ((humans[Filter]) AND (english[Filter]))

Similar strategies, adapted to the syntax of each database, were applied to Scopus, Science Direct, and the Cochrane Library.

S2: Forest Plots of Subgroup Analysis Results

A) **By type of cohort:**

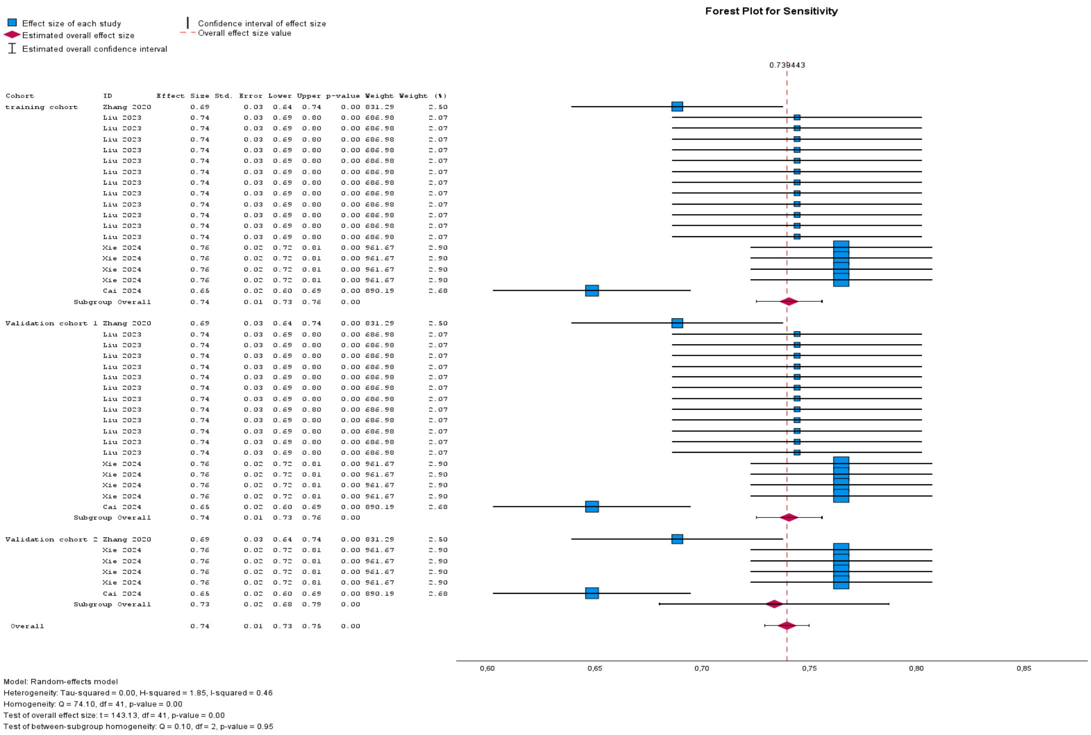

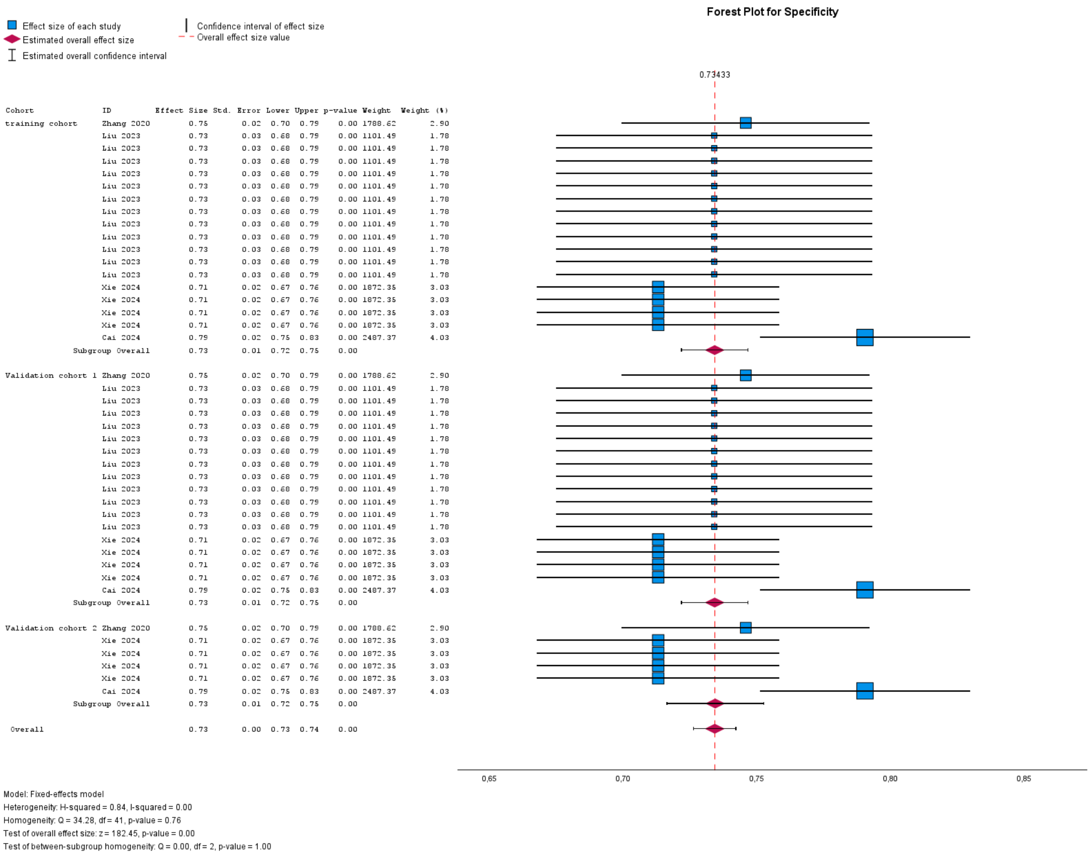

Figure S1. Forest plot demonstrating the pooled diagnostic performance (sensitivity and specificity) of CT radiomics in predicting the Ki-67 index of GISTs, derived from a subgroup analysis segmented by individual study cohorts.Studies are listed in chronological order.

B) By CT imaging protocols

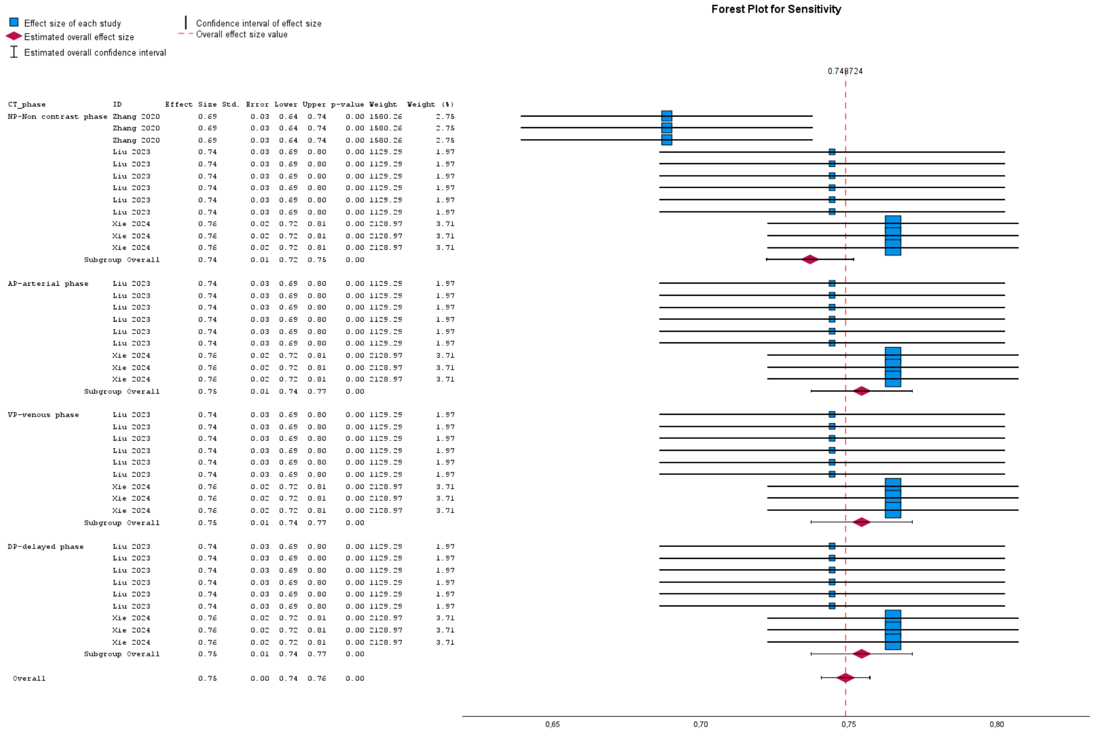

Model: Fixed-effects model
Heterogeneity: H-squared = 0.64, I-squared = 0.00
Homogeneity: Q = 24.41, df = 38, p-value = 0.96
Test of overall effect size: z = 179.37, p-value = 0.00
Test of between-subgroup homogeneity: Q = 3.74, df = 3, p-value = 0.29

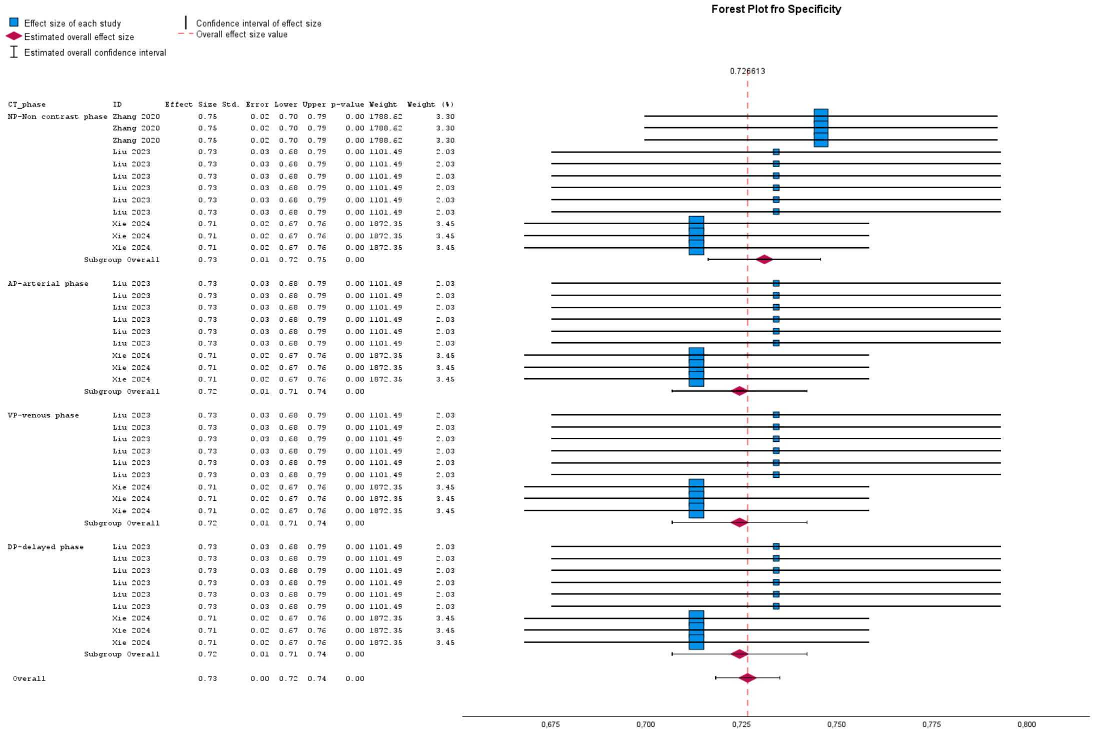

Model: Fixed-effects model
Heterogeneity: H-squared = 0.20, I-squared = 0.00
Homogeneity: Q = 7.54, df = 38, p-value = 1.00
Test of overall effect size: z = 169.27, p-value = 0.00
Test of between-subgroup homogeneity: Q = 0.51, df = 3, p-value = 0.92

Figure S2. Forest plot demonstrating the pooled diagnostic performance (sensitivity and specificity) of CT radiomics in predicting the Ki-67 index of GISTs, derived from a subgroup analysis segmented by individual study CT imaging protocols.Studies are listed in chronological order.

C) By the Number of Radiomics Features

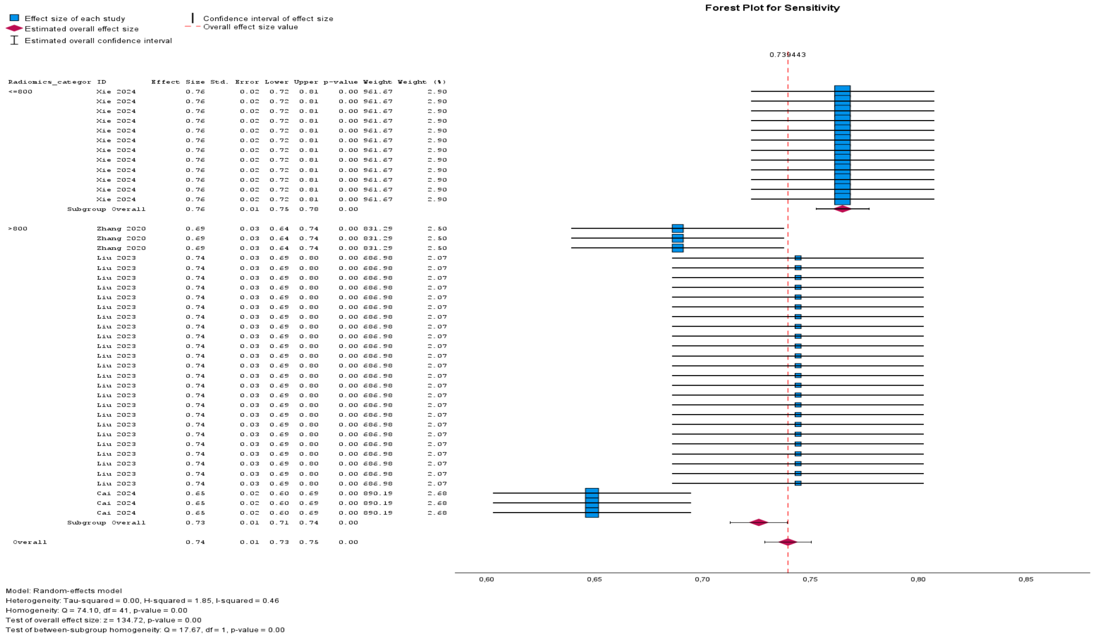

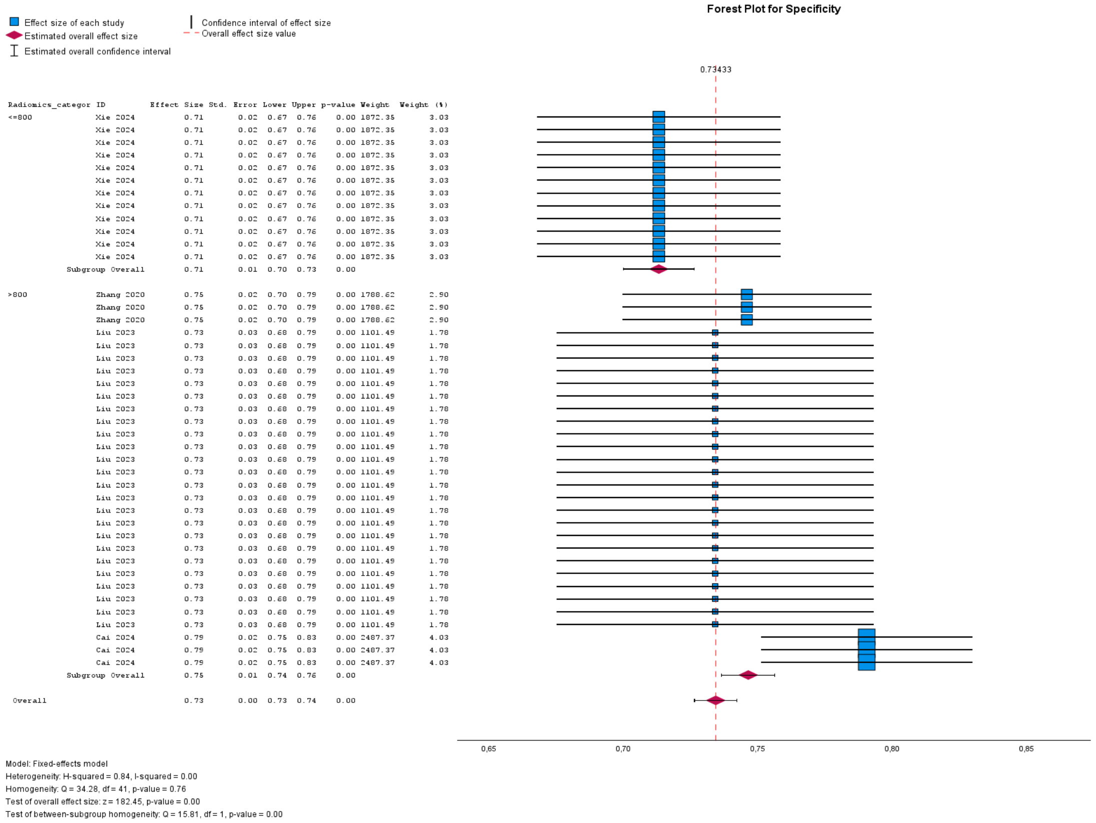

Figure S3. Forest plot demonstrating the pooled diagnostic performance (sensitivity and specificity) of CT radiomics in predicting the Ki-67 index of GISTs, derived from a subgroup analysis segmented by individual study number of radiomics features.Studies are listed in chronological order.

D) By the Ki-67 Index Cutoff

Forest Plot for Sensitiivity

Effect size of each study
Estimated overall effect size
Estimated overall confidence interval
Confidence interval of effect size
Overall effect size value

Ki67_cutoff	ID	Effect Size	Std. Error	Lower	Upper	p-value	Weight	Weight (%)
8	Cai 2024	0.65	0.02	0.60	0.69	0.00	890.19	2.68
	Cai 2024	0.65	0.02	0.60	0.69	0.00	890.19	2.68
	Cai 2024	0.65	0.02	0.60	0.69	0.00	890.19	2.68
	Subgroup Overall	0.65	0.01	0.62	0.68	0.00		
10	Zhang 2020	0.69	0.03	0.64	0.74	0.00	831.29	2.50
	Zhang 2020	0.69	0.03	0.64	0.74	0.00	831.29	2.50
	Zhang 2020	0.69	0.03	0.64	0.74	0.00	831.29	2.50
	Liu 2023	0.74	0.03	0.69	0.80	0.00	686.98	2.07
	Liu 2023	0.74	0.03	0.69	0.80	0.00	686.98	2.07
	Liu 2023	0.74	0.03	0.69	0.80	0.00	686.98	2.07
	Liu 2023	0.74	0.03	0.69	0.80	0.00	686.98	2.07
	Liu 2023	0.74	0.03	0.69	0.80	0.00	686.98	2.07
	Liu 2023	0.74	0.03	0.69	0.80	0.00	686.98	2.07
	Liu 2023	0.74	0.03	0.69	0.80	0.00	686.98	2.07
	Liu 2023	0.74	0.03	0.69	0.80	0.00	686.98	2.07
	Liu 2023	0.74	0.03	0.69	0.80	0.00	686.98	2.07
	Liu 2023	0.74	0.03	0.69	0.80	0.00	686.98	2.07
	Liu 2023	0.74	0.03	0.69	0.80	0.00	686.98	2.07
	Liu 2023	0.74	0.03	0.69	0.80	0.00	686.98	2.07
	Liu 2023	0.74	0.03	0.69	0.80	0.00	686.98	2.07
	Liu 2023	0.74	0.03	0.69	0.80	0.00	686.98	2.07
	Liu 2023	0.74	0.03	0.69	0.80	0.00	686.98	2.07
	Liu 2023	0.74	0.03	0.69	0.80	0.00	686.98	2.07
	Liu 2023	0.74	0.03	0.69	0.80	0.00	686.98	2.07
	Liu 2023	0.74	0.03	0.69	0.80	0.00	686.98	2.07
	Liu 2023	0.74	0.03	0.69	0.80	0.00	686.98	2.07
	Liu 2023	0.74	0.03	0.69	0.80	0.00	686.98	2.07
	Liu 2023	0.74	0.03	0.69	0.80	0.00	686.98	2.07
	Liu 2023	0.74	0.03	0.69	0.80	0.00	686.98	2.07
	Liu 2023	0.74	0.03	0.69	0.80	0.00	686.98	2.07
	Liu 2023	0.74	0.03	0.69	0.80	0.00	686.98	2.07
	Xie 2024	0.76	0.02	0.72	0.81	0.00	961.67	2.90
	Xie 2024	0.76	0.02	0.72	0.81	0.00	961.67	2.90
	Xie 2024	0.76	0.02	0.72	0.81	0.00	961.67	2.90
	Xie 2024	0.76	0.02	0.72	0.81	0.00	961.67	2.90
	Xie 2024	0.76	0.02	0.72	0.81	0.00	961.67	2.90
	Xie 2024	0.76	0.02	0.72	0.81	0.00	961.67	2.90
	Xie 2024	0.76	0.02	0.72	0.81	0.00	961.67	2.90
	Xie 2024	0.76	0.02	0.72	0.81	0.00	961.67	2.90
	Xie 2024	0.76	0.02	0.72	0.81	0.00	961.67	2.90
	Xie 2024	0.76	0.02	0.72	0.81	0.00	961.67	2.90
	Xie 2024	0.76	0.02	0.72	0.81	0.00	961.67	2.90
	Xie 2024	0.76	0.02	0.72	0.81	0.00	961.67	2.90
	Subgroup Overall	0.75	0.00	0.74	0.76	0.00		
Overall		0.74	0.01	0.73	0.75	0.00		

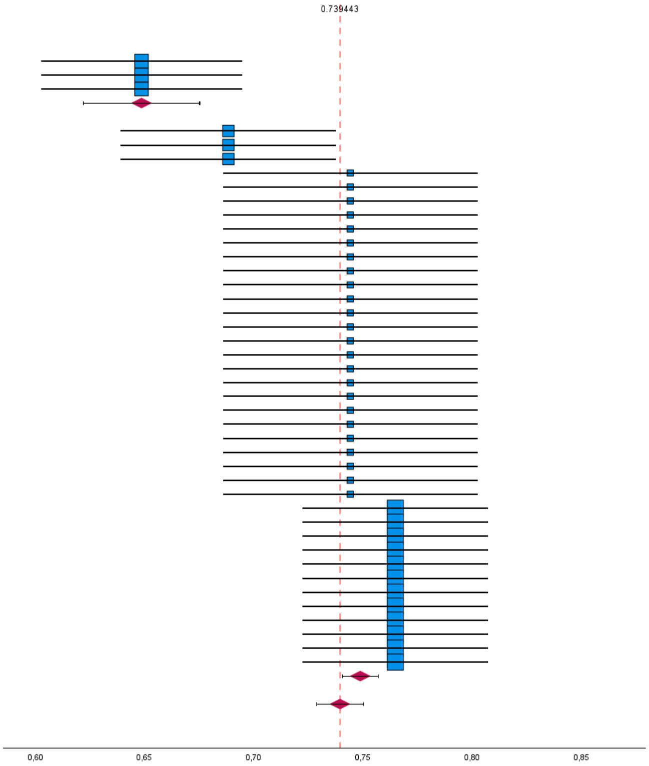

Model: Random-effects model
Heterogeneity: Tau-squared = 0.00, H-squared = 1.85, I-squared = 0.46
Homogeneity: Q = 74.10, df = 41, p-value = 0.00
Test of overall effect size: z = 134.72, p-value = 0.00
Test of between-subgroup homogeneity: Q = 49.69, df = 1, p-value = 0.00

Forest Plot for Specificity

Effect size of each study
Estimated overall effect size
Estimated overall confidence interval
Confidence interval of effect size
Overall effect size value

Ki67_cutoff	ID	Effect Size	Std. Error	Lower	Upper	p-value	Weight	Weight (%)
8	Cai 2024	0.79	0.02	0.75	0.83	0.00	2487.37	4.03
	Cai 2024	0.79	0.02	0.75	0.83	0.00	2487.37	4.03
	Cai 2024	0.79	0.02	0.75	0.83	0.00	2487.37	4.03
	Subgroup Overall	0.79	0.01	0.77	0.81	0.00		
10	Zhang 2020	0.75	0.02	0.70	0.79	0.00	1788.62	2.90
	Zhang 2020	0.75	0.02	0.70	0.79	0.00	1788.62	2.90
	Zhang 2020	0.75	0.02	0.70	0.79	0.00	1788.62	2.90
	Liu 2023	0.73	0.03	0.68	0.79	0.00	1101.49	1.78
	Liu 2023	0.73	0.03	0.68	0.79	0.00	1101.49	1.78
	Liu 2023	0.73	0.03	0.68	0.79	0.00	1101.49	1.78
	Liu 2023	0.73	0.03	0.68	0.79	0.00	1101.49	1.78
	Liu 2023	0.73	0.03	0.68	0.79	0.00	1101.49	1.78
	Liu 2023	0.73	0.03	0.68	0.79	0.00	1101.49	1.78
	Liu 2023	0.73	0.03	0.68	0.79	0.00	1101.49	1.78
	Liu 2023	0.73	0.03	0.68	0.79	0.00	1101.49	1.78
	Liu 2023	0.73	0.03	0.68	0.79	0.00	1101.49	1.78
	Liu 2023	0.73	0.03	0.68	0.79	0.00	1101.49	1.78
	Liu 2023	0.73	0.03	0.68	0.79	0.00	1101.49	1.78
	Liu 2023	0.73	0.03	0.68	0.79	0.00	1101.49	1.78
	Liu 2023	0.73	0.03	0.68	0.79	0.00	1101.49	1.78
	Liu 2023	0.73	0.03	0.68	0.79	0.00	1101.49	1.78
	Liu 2023	0.73	0.03	0.68	0.79	0.00	1101.49	1.78
	Liu 2023	0.73	0.03	0.68	0.79	0.00	1101.49	1.78
	Liu 2023	0.73	0.03	0.68	0.79	0.00	1101.49	1.78
	Liu 2023	0.73	0.03	0.68	0.79	0.00	1101.49	1.78
	Liu 2023	0.73	0.03	0.68	0.79	0.00	1101.49	1.78
	Liu 2023	0.73	0.03	0.68	0.79	0.00	1101.49	1.78
	Liu 2023	0.73	0.03	0.68	0.79	0.00	1101.49	1.78
	Liu 2023	0.73	0.03	0.68	0.79	0.00	1101.49	1.78
	Liu 2023	0.73	0.03	0.68	0.79	0.00	1101.49	1.78
	Liu 2023	0.73	0.03	0.68	0.79	0.00	1101.49	1.78
	Xie 2024	0.71	0.02	0.67	0.76	0.00	1872.35	3.03
	Xie 2024	0.71	0.02	0.67	0.76	0.00	1872.35	3.03
	Xie 2024	0.71	0.02	0.67	0.76	0.00	1872.35	3.03
	Xie 2024	0.71	0.02	0.67	0.76	0.00	1872.35	3.03
	Xie 2024	0.71	0.02	0.67	0.76	0.00	1872.35	3.03
	Xie 2024	0.71	0.02	0.67	0.76	0.00	1872.35	3.03
	Xie 2024	0.71	0.02	0.67	0.76	0.00	1872.35	3.03
	Xie 2024	0.71	0.02	0.67	0.76	0.00	1872.35	3.03
	Xie 2024	0.71	0.02	0.67	0.76	0.00	1872.35	3.03
	Xie 2024	0.71	0.02	0.67	0.76	0.00	1872.35	3.03
	Xie 2024	0.71	0.02	0.67	0.76	0.00	1872.35	3.03
	Xie 2024	0.71	0.02	0.67	0.76	0.00	1872.35	3.03
	Subgroup Overall	0.73	0.00	0.72	0.74	0.00		
Overall		0.73	0.00	0.73	0.74	0.00		

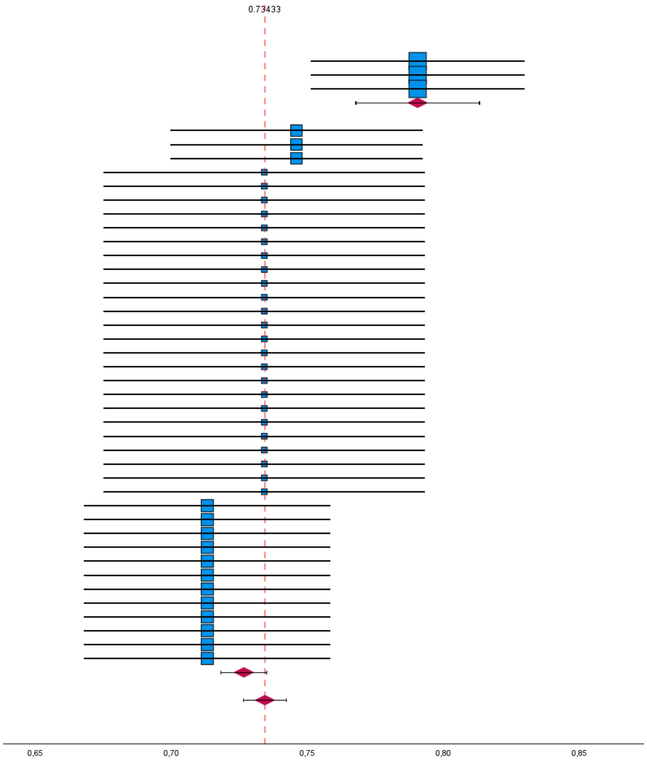

Model: Fixed-effects model
Heterogeneity: H-squared = 0.84, I-squared = 0.00
Homogeneity: Q = 34.28, df = 41, p-value = 0.76
Test of overall effect size: z = 182.45, p-value = 0.00
Test of between-subgroup homogeneity: Q = 26.74, df = 1, p-value = 0.00

Figure S4. Forest plot demonstrating the pooled diagnostic performance (sensitivity and specificity) of CT radiomics in predicting the Ki-67 index of GISTs, derived from a subgroup analysis segmented by individual study Ki-67 index cutoff. Studies are listed in chronological order.
