# Supplementary material for: Can CT Radiomics Predict the Ki-67 Index of Gastrointestinal Stromal Tumors (GISTs)? A Systematic Review and Meta-Analysis
Source: Cancers (Basel). 2025 Aug 30;17(17):2855. doi: 10.3390/cancers17172855 (PMC12427292; doi:10.3390/cancers17172855)
Supplement: Supplementary file 1 [file cancers-17-02855-s001.zip › Take home message.pdf]

Take home-message

• Six studies (1632 patients) were included in this systematic review and meta-analysis.
• CT radiomics achieved moderate diagnostic accuracy for predicting Ki-67 index in gastrointestinal stromal tumors (GISTs), with pooled sensitivity of 0.71, specificity of 0.76, and AUC of 0.79.
• Findings were consistent across imaging protocols and feature sets, but diagnostic performance varied with different Ki-67 cutoffs (8% vs. 10%).
• Radiomics provides a non-invasive alternative to biopsy, capturing whole-tumor heterogeneity and supporting preoperative risk stratification.
• Limitations remain due to study heterogeneity, retrospective designs, and lack of standardization.
• Future directions: multicenter prospective studies, standardized radiomics pipelines, and integration with genomic/clinical data to guide personalized therapy, including immunotherapy.
